# Design and Analysis of Joint Group Shuffled Scheduling Decoding Algorithm for Double LDPC Codes System

**DOI:** 10.3390/e25020357

**Published:** 2023-02-15

**Authors:** Qiwang Chen, Yanzhao Ren, Lin Zhou, Chen Chen, Sanya Liu

**Affiliations:** Xiamen Key Laboratory of Mobile Multimedia Communications, College of Information Science and Engineering, Huaqiao University, Xiamen 361021, China

**Keywords:** joint source-channel coding, shuffled scheduling decoding, belief propagation, EXIT, LDPC code

## Abstract

In this paper, a joint group shuffled scheduling decoding (JGSSD) algorithm for a joint source-channel coding (JSCC) scheme based on double low-density parity-check (D-LDPC) codes is presented. The proposed algorithm considers the D-LDPC coding structure as a whole and applies shuffled scheduling to each group; the grouping relies on the types or the length of the variable nodes (VNs). By comparison, the conventional shuffled scheduling decoding algorithm can be regarded as a special case of this proposed algorithm. A novel joint extrinsic information transfer (JEXIT) algorithm for the D-LDPC codes system with the JGSSD algorithm is proposed, by which the source and channel decoding are calculated with different grouping strategies to analyze the effects of the grouping strategy. Simulation results and comparisons verify the superiority of the JGSSD algorithm, which can adaptively trade off the decoding performance, complexity and latency.

## 1. Introduction

In an integrated communication system [1], Shannon’s separation principle indicates that arbitrarily high reliability can be attained for infinite source and channel code block lengths. In a nonasymptotic regime, a joint source-channel coding (JSCC) [2] design can be more attractive, allowing source redundancy and channel information to be exchanged iteratively to improve decoding performance. In addition, the JSCC system can also reduce decoding complexity and transmission delay, which results in its successful application in image and video transmission [3,4].

A JSCC scheme based on double low-density parity-check (D-LDPC) codes was proposed [2]; in this scheme, one LDPC is for source compression and another is for channel error-control. As the LDPC code can be represented by a Tanner graph, a belief propagation (BP) decoding algorithm can be applied. The source and channel coding structures both perform BP decoding, pass decoding information to each other and accomplish information exchange. However, this is a parallel decoding method and needs multiple pieces of calculation hardware working simultaneously. This high decoding complexity is not suitable for low-complexity green communication systems, e.g., Internet of Things (IoT) [5].

### 1.1. Related Work and Motivation of Shuffled Scheduling Decoding

Recently, the concept of shuffled scheduling decoding [6], a kind of serial decoding, was introduced into the D-LDPC codes system [7]. This can lower the decoding hardware complexity and reduce the number of decoding iterations (an iteration indicates all of the VNs and CNs being updated one time). In order to take full advantage of the linking relationship, a more generalized shuffled scheduling decoding algorithm [8] was proposed. However, this algorithm respectively applied the shuffled scheduling strategy to source decoding and channel decoding, which in fact is a separated decoding method, denoted as the separated shuffled scheduling decoding (SSSD) algorithm. Compared with the conventional BP decoding algorithm, the SSSD algorithm has a higher decoding latency.

In this paper, a joint shuffled scheduling decoding algorithm will be proposed for the D-LDPC codes system from a global viewpoint. The proposed algorithm is the generalization version of the SSSD algorithm. It considers the Tanner graph of source and channel coding structure as a whole and applies shuffled decoding. Moreover, the joint Tanner graph can be divided into a number of groups to trade off the decoding performance and decoding latency. The grouping relies on the types or on the code-length. We describe the decoding algorithm as a joint group shuffled scheduling decoding (JGSSD) algorithm. Specifically, if the grouping divides the Tanner graph into two parts, i.e., the source and channel parts, then the JGSSD algorithm changes to the SSSD algorithm.

### 1.2. Related Work on D-LDPC Codes Systems

In recent years, a large amount of research has focused on the investigation of the D-LDPC codes system [9,10,11]. The main difference between the D-LDPC system and single channel LDPC code systems is the consideration of nonuniform sources and source coding. For the D-LDPC codes system, the source coding may cause the loss of original information and trigger the phenomenon of error floor. In order to improve the performance of the error floor region, a linking matrix is set up between the variable nodes (VNs) of the source code and the check nodes (CNs) of the channel codes [12], and more original information participates in coding. The improvement of the error floor can be evaluated by the source decoding threshold and analyzed by the source protograph EXIT (SPEXIT) algorithm [13]. The linking matrix is further optimized for high-entropy sources [14]. The source LDPC coding matrix can be also optimized to match the source statistic [15] as well as the joint optimization of the source coding matrix and linking matrix [16].

On the other hand, the source redundancy left in the source coding affects the specific structure of the D-LDPC codes. Firstly, the effect of the source statistic is analyzed over the Rayleigh fading channel compared with reception diversity [17]. The channel decoding threshold can be evaluated using the joint protograph EXIT (JPEXIT) algorithm, by which the channel P-LDPC codes [18] and the allocation [19,20] of an important structure, i.e., degree-2 VNs [21], are redesigned. Several works are also performed for the joint component design, including the optimized source and channel pairs [22] and the joint coding matrix [23,24].

In addition, an information shortening strategy was conducted to reduce the effects of the short cycles in the Tanner graph [25]. The D-LDPC codes system was also considered in some nonstandard coding channels [26,27,28]. Spatially coupled LDPC codes were introduced into the D-LDPC codes system [29]; these can perform sliding window decoding (SWD) for significantly reduced latency and complexity requirements. A proposed SWD algorithm [30] with variable window size was optimized for balancing performance and complexity. The D-LDPC codes system has been applied to image transmission [31,32].

### 1.3. Main Contribution

The aforementioned D-LDPC codes systems mostly perform BP decoding algorithms. In this paper, a joint decoding viewpoint is introduced, and shuffled scheduling decoding for the D-LDPC codes system is generalized.

The novelty and contributions of this paper can be summarized as follows:

(1) From a global viewpoint, the D-LDPC codes structure is considered as a whole, and a joint shuffled scheduling decoding strategy is introduced to the D-LDPC codes system.
(2) A grouping method for the joint shuffled scheduling decoding strategy, which relies on the types or the length of the VNs, is introduced.
(3) A novel EXIT algorithm to calculate the channel and source decoding thresholds for the general D-LDPC coding structure with the JGSSD algorithm is proposed.
(4) A comparison between the SSSD algorithm and the JGSSD algorithm is conducted, including decoding performance, decoding complexity and decoding latency.

The main differences between the present work and previous work are shown in Table 1.

### 1.4. Paper Organization

The remainder of the paper is organized as follows. In Section 2, we describe the preliminaries of the D-LDPC codes. The joint shuffled scheduling decoding algorithm with grouping strategy is proposed in Section 3. An EXIT algorithm for analyzing the D-LDPC codes system with JGSSD algorithm is presented in Section 4. A simulation and comparisons are conducted in Section 5. Finally, Section 6 draws the conclusions of this paper.

## 2. Preliminaries of D-LDPC Codes Systems

### 2.1. The D-LDPC Coding Structure

An LDPC code can be represented by a protograph, a small protomatrix $B=[b_{ij}]$, where $b_{ij}$ indicates the number of edges connecting a VN $v_{j}$ to a CN $c_{i}$. Then, a large parity-check matrix can be obtained by a “copy-and-permute” operation, such as the progressive edge growth (PEG) algorithm [33] with a lifting factor.

A D-LDPC code can be represented by a joint protograph $B_{J}$ as follows:

(1) $$\begin{array}{c} B_{J}=\left[\begin{array}{cc} B_{s} & B_{l1}\\ B_{l2} & B_{c} \end{array}\right], \end{array}$$

where $B_{s}$ is the source coding protomatrix of size $m_{s}\times n_{s}$, $B_{c}$ is the channel coding protomatrix of size $m_{c}\times n_{c}$, $B_{l1}=\left[I\;0\right]$ (I is an identity matrix) is the source-check-channel-variable linking protomatrix of size $m_{s}\times n_{c}$ and $B_{l2}$ is the source-variable-channel-check linking protomatrix of size $m_{c}\times n_{s}$. Then, a joint parity-check matrix $H_{J}$ can be derived:

(2) $$\begin{array}{c} H_{J}=\left[\begin{array}{cc} H_{s} & H_{l1}\\ H_{l2} & H_{c} \end{array}\right], \end{array}$$

where $H_{s}$ is the source coding matrix of size $M_{s}\times N_{s}$, $H_{c}$ is the channel coding matrix of size $M_{c}\times N_{c}$, $H_{l1}=\left[I\;0\right]$ is the source-check-channel-variable linking matrix of size $M_{s}\times N_{c}$ and $H_{l2}$ is the source-variable-channel-check linking matrix of size $M_{c}\times N_{s}$. The overall code rate of the D-LDPC codes is given by $R_{overall}=\frac{N_{s}}{M_{s}}\times \frac{N_{c}-M_{c}}{N_{c}}$.

### 2.2. Transmission System Model

Assume that original source bits $s\in {\left\{0,1\right\}}^{\left(1\times N_{s}\right)}$ are generated from a binary independent and identically distributed (i.i.d) Bernoulli source, where the probability of “1” is η. The encoding procedures with a nonzero $H_{l2}$ are given as follows. Firstly, the compressed source bits c can be obtained by

(3) $$\begin{array}{c} c=s{\left(H_{s}\right)}^{T}, \end{array}$$

where ${\left(\cdot \right)}^{T}$ represents the matrix transposition in math. Then, a codeword u can be obtained by

(4) $$\begin{array}{c} u=\left[s,c\right]G_{p}, \end{array}$$

where $G_{p}$ is a systematic generator matrix obtained from $\left[H_{l2}\;H_{c}\right]$. Thus, the u consists of three parts, i.e., u=[s,c,p], where p is the parity bits. Finally, the channel codeword d=[c,p] is sent to the channel, after puncturing if punctured LDPC code is considered. If $H_{l2}=0$, the encoding procedure can be simplified by $d=\left[c,p\right]=s{\left(H_{s}\right)}^{T}G_{c}$, where $G_{c}$ is obtained from $H_{c}$.

**Figure 1:**
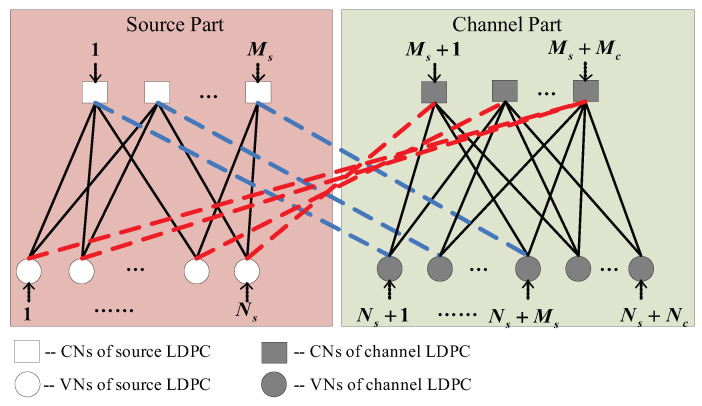
The joint Tanner graph of the D-LDPC codes system.

In the decoding process, a binary-phase-shift keying (BPSK) and AWGN channel model are assumed and the log-likelihood-ratio (LLR) values of all VNs are first initialized. Then, as shown in Figure 1, the iterative BP (IBP) algorithm is performed as follows:

(1) Update all the C2V messages for each of the $M_{c}$ CNs in the channel part;
(2) Update all the V2C messages for each of the $N_{s}$ VNs in the source part;
(3) Update all the C2V messages for each of the $M_{s}$ CNs in the source part;
(4) Update all the V2C messages for each of the $N_{c}$ VNs in the channel part;
(5) The source part and channel part exchange decoding information through $H_{l1}$ and $H_{l2}$ (i.e., the dashed blue and red lines in Figure 1);
(6) Estimate the codeword $\hat{u}$ based on the posterior LLRs at the VNs;
(7) Repeat Steps (1) to (6), unless (i) the estimated codeword $\hat{s}$ and $\hat{d}$ satisfy $\hat{s}{\left(H_{s}\right)}^{T}=0$ and $\hat{d}{\left(H_{c}\right)}^{T}=0$ (ii) the maximum iteration number *K* is reached.

For the SSSD algorithm in [7,8], the updates for C2V in the channel part and source part, i.e., Step (1) and (3), are performed using shuffled scheduling. For more details about the decoding procedure, the reader can refer to [8].

## 3. Joint Group Shuffled Scheduling Decoding Algorithm

It can be observed that the BP and SSSD algorithms mentioned above are both types of iterative decoding method between the source decoder and channel decoder. The shuffled scheduling decoding is only respectively applied in source decoding and channel decoding, and not applied to the update of $H_{l1}$ and $H_{l2}$. Thus, the SSSD algorithm is in fact a separated decoding method.

Here, considering that the vector u satisfies

(5) $$\begin{array}{cc} u{\left(H_{J}\right)}^{T} & =\left[s,c,p\right]{\left(H_{J}\right)}^{T}\\ \; & =\left[s,c,p\right]{\left[\begin{array}{cc} H_{s} & I\;0\\ H_{l2} & H_{c} \end{array}\right]}^{T}\\ \; & =\left[s,c,p\right]\left[\begin{array}{cc} {\left(H_{s}\right)}^{T} & 0\\ \begin{array}{c} {\left(I\right)}^{T}\\ 0 \end{array} & {\left(H_{c}\right)}^{T} \end{array}\right]\\ \; & =\left[\left[s,c\right]{\left[\begin{array}{c} H_{s}\\ I \end{array}\right]}^{T},\left[c,p\right]{\left(H_{c}\right)}^{T}\right], \end{array}$$

where $\left[s,c\right]{\left[\begin{array}{c} H_{s}\\ I \end{array}\right]}^{T}=s{\left(H_{s}\right)}^{T}+c=c+c=0$ and $\left[c,p\right]{\left(H_{c}\right)}^{T}=0$, so that $u{\left(H_{J}\right)}^{T}=0$.

Thus, the combined Tanner graph of the source part and the channel part can be considered a joint Tanner graph. We apply the BP decoding to the D-LDPC codes system, and this is denoted as a joint BP decoding (JBP) algorithm. For ease of description of the JBP algorithm, several types of LLRs are defined, and *k*-th iteration is assumed.

- $z_{n}^{s}$ represents the LLR of the *n*-th bit of original source s.
- $z_{n}^{d}$ represents the LLR of the *n*-th bit of codeword d.
- $\varepsilon_{mn}^{k}$ represents the LLR from the *m*-th CN to the *n*-th VN at *k*-th iteration.
- $\phi_{mn}^{k}$ represents the LLR from the *n*-th VN to the *m*-th CN at *k*-th iteration.
- $\Phi_{n}^{k}$ represents the LLRs of the *n*-th bit at *k*-th iteration.

Based on the above definitions, the decoding procedure is described as follows.

**Initialization:** The initial LLR of VNs can be calculated by

(6) $$\begin{array}{c} z_{n}^{s}=\ln((1-\eta )/\eta ),\left(n=1,2,\cdots ,N_{s}\right) \end{array}$$

and

(7) $$\begin{array}{c} z_{n}^{d}=\left(2r_{n}\right)/\sigma^{2},\left(n=1,2,\cdots ,N_{c}\right), \end{array}$$

where $r_{n}$ is the *n*-th received signal and $\sigma^{2}$ is the noise variance, and the LLRs are 0 for the punctured bits. Furthermore, the $\sigma^{2}$ can be calculated by $\sigma^{2}=1/\left(2\times R_{overall}\times \left(E_{s}/N_{0}\right)\right)$, where $E_{s}$ is the average transmitted energy per source information bit, and $N_{0}$ is the noise power spectral density.

**Step 1:** Update C2V messages, for $1\leq n\leq N_{s}+N_{c}$ and $1\leq m\leq M_{s}+M_{c}$,

(8) $$\begin{array}{c} \varepsilon_{mn}^{k}=2\tanh^{-1}\left(\prod_{\overset{n^{\prime }\neq n}{}}\tanh\left(\frac{\phi_{mn^{\prime }}^{k-1}}{2}\right)\right). \end{array}$$

**Step 2:** Update V2C messages, for $1\leq n\leq N_{s}+N_{c}$ and $1\leq m\leq M_{s}+M_{c}$,

(9) $$\begin{array}{c} \phi_{mn}^{k}=\left\{\begin{array}{cc} z_{n}^{s}+\sum_{m^{\prime }\in \Theta \left(n\right)\backslash m}\varepsilon_{m^{\prime }n}^{k}, & \mathrm{if}\;1\leq n\leq N_{s}\\ z_{n}^{d}+\sum_{m^{\prime }\in \Theta \left(n\right)\setminus m}\varepsilon_{m^{\prime }n}^{k}, & \mathrm{if}\;N_{s}+1\leq n\leq N_{s}+N_{c} \end{array}\right. \end{array}$$

and

(10) $$\begin{array}{c} \Phi_{n}^{k}=\left\{\begin{array}{cc} z_{n}^{s}+\sum_{m\in \Theta (n)}\varepsilon_{mn}^{k}, & \mathrm{if}\;1\leq n\leq N_{s}\\ z_{n}^{d}+\sum_{m\in \Theta (n)}\varepsilon_{mn}^{k}, & \mathrm{if}\;N_{s}+1\leq n\leq N_{s}+N_{c} \end{array}\right.. \end{array}$$

where Θ(n) denotes all CNs connected to the *n*-th VN. For $1\leq n\leq N_{s}+N_{c}$, if $\Phi_{n}^{k}\geq 0$, ${\hat{u}}_{n}=0$; otherwise, ${\hat{u}}_{n}=1$, where $\hat{u}=\left[{\hat{u}}_{n}\right]\left(n=1,\cdots ,N_{s}+N_{c}\right)$ is the estimated bit codeword.

**Step 3:** Stopping condition: if $\hat{u}\cdot H_{J}=0$ or $k=K_{max}$, the iteration will stop; otherwise, set k=k+1 and go to Step 1, where $K_{max}$ is the maximum iteration of decoding.

For the joint shuffled scheduling BP algorithm, the initialization and stopping conditions remain the same as in the JBP algorithm. The only difference between the two algorithms lies in the updating procedure. For the updated C2V message, certain $\phi_{mn^{\prime }}^{k}$ have been updated in Step 2 and can be used instead of $\phi_{mn^{\prime }}^{k-1}$ in Step 1 to calculate the remaining values $\varepsilon_{mn}^{k}$. Thus, the updated C2V message can be modified as follows:

(11) $$\begin{array}{c} \varepsilon_{mn}^{k}=2\tanh^{-1}\left(\prod_{\overset{n^{\prime }<n}{}}\tanh\left(\frac{\phi_{mn^{\prime }}^{k}}{2}\right)\times \prod_{\overset{n^{\prime }>n}{}}\tanh\left(\frac{\phi_{mn^{\prime }}^{k-1}}{2}\right)\right). \end{array}$$

However, it is observed that one iteration of the standard JBP algorithm can be fully processed in parallel, while that of a shuffled JBP algorithm becomes totally serial, and this will bring about a large decoding latency. To decrease decoding latency and keep the parallelism advantages of the standard JBP, the concept of grouping is introduced, and the decoding algorithm is developed into a joint group shuffled scheduling decoding (JGSSD) algorithm. Before performing BP decoding, the decoding information is first divided into a certain number of groups. The updating of information in each group is processed in parallel, but the processing of groups remains serial. In detail, the VNs are divided into a number of groups according to certain criteria, i.e.,

(12) $$\begin{array}{c} V=\{V^{1},V^{2},\cdots ,V^{G}\}, \end{array}$$

where $V^{g}=\left[V_{i}^{g}\right]\left(g=1,2,\cdots ,G^{H},i=1,2,\cdots ,n_{g}\right)$, $G^{H}$ is the number of groups and $n_{g}$ is the size of $V^{g}$. Thus, the updated C2V message can be modified as follows:

For $n\in V^{g}$, i.e., $N_{g-1}<n<N_{g-1}+n_{g}$ and m∈Θ(n),

(13) $$\begin{array}{c} \varepsilon_{mn}^{k}=2\tanh^{-1}\left(\prod_{\overset{n^{\prime }\in \Theta \left(m\right)\backslash n}{n^{\prime }⩽N_{g-1}}}\tanh\left(\frac{\phi_{mn^{\prime }}^{k}}{2}\right)\times \prod_{\overset{n^{\prime }\in \Theta \left(m\right)\backslash n}{n^{\prime }>N_{g-1}}}\tanh\left(\frac{\phi_{mn^{\prime }}^{k-1}}{2}\right)\right), \end{array}$$

where Θ(m) denotes all VNs connected to the *m*-th CN, Θ(n) denotes all CNs connected to the *n*-th VN, Θ(m)\n denotes all VNs in Θ(m) excluding the *n*-th VN, $N_{g-1}$ is the size of the sum of the former sets $\left\{V^{1},V^{2},\cdots ,V^{g-1}\right\}$.

For the JGSSD algorithm, we can group the V based on code length. For example, we assumed that the $N_{s}+N_{c}$ VNs are divided into $G^{H}$ groups on average, and that each group contains $\left(N_{s}+N_{c}\right)/G^{H}$ VNs (assuming $\left(N_{s}+N_{c}\right)$ mod $G^{H}=0$ for simplicity). We can also group the V according to the types of VNs. For example, $V=\{V^{1},V^{2}\}$ and $V^{1}=\left\{\mathrm{source}\;\mathrm{VNs}\right\}$ of length $N_{s}$ and $V^{2}=\left\{\mathrm{channel}\;\mathrm{VNs}\right\}$ of length $N_{c}$. Now, the JGSSD becomes the IBP algorithm. If the $V^{1}$ and $V^{2}$ are further divided into $N_{s}$ and $N_{c}$ groups, respectively, this grouping strategy makes the algorithm change to be the SSSD version.

## 4. Analysis of the D-LDPC Codes System with JGSSD Algorithm

### 4.1. Joint Shuffled Extrinsic Information Algorithm

EXIT analysis can reflect the ultimate performance of a D-LDPC codes system by calculating the decoding threshold of its corresponding protograph. Although an EXIT algorithm for analyzing the decoding threshold of the D-LDPC codes system with shuffled scheduling has been proposed in [34], it only aimed at the $B_{J}$ with $B_{l2}=0$. In addition, the EXIT algorithm is comprised of source and channel parts, just like the decoding procedure. Thus, it is not suitable for the D-LDPC codes system with general structure and JGSSD algorithm. In this section, a joint shuffled extrinsic information transfer (JSEXIT) algorithm is proposed.

Firstly, five types of mutual information (MI) are defined as:

- $I_{ij,(k)}^{Ev}$: the extrinsic MI from *j*-th VN to *i*-th CN at *k*-th iteration;
- $I_{ij,(k)}^{Ec}$: the extrinsic MI from *i*-th CN to *j*-th VN at *k*-th iteration;
- $I_{ij,(k)}^{Av}$: the a priori MI from *j*-th VN to *i*-th CN at *k*-th iteration;
- $I_{ij,(k)}^{Ac}$: the a priori MI from *i*-th CN to *j*-th VN at *k*-th iteration;
- $I_{j,(k)}^{APP}$: the MI between a posteriori LLR evaluated by *j*-th VN and the corresponding source bit $s_{j}$ at *k*-th iteration.

In addition, an indicator function is defined as follows:

(14) $$\Omega \left(b_{ij}\right)=\left\{\begin{array}{cc} 1 & \mathrm{if}\;b_{ij}\neq 0\\ 0 & \mathrm{otherwise} \end{array}\right.$$

and if a VN is punctured, its initial LLR value is 0. Moreover, $J(\sigma_{ch})$ represents the MI between a binary bit and its corresponding LLR value $L_{ch}∼N\left(\sigma_{ch}^{2}/2,\sigma_{ch}^{2}\right)$$N(\theta ,\sigma^{2}$) represent the Gaussian distribution with expectation θ and variance $\sigma^{2}$, given by [2]

(15) $$J\left(\sigma_{ch}\right)=1-\int_{-\infty }^{\infty }\frac{e^{-{\left(\xi -\sigma_{ch}^{2}/2\right)}^{2}/2\sigma_{ch}^{2}}}{\sqrt{2\pi \sigma_{ch}^{2}}}\cdot \log_{2}\left(1+e^{-\xi }\right)d\xi .$$

Then, the VNs of the joint protograph are divided into a number of groups according to certain criteria, i.e.,

(16) $$\begin{array}{c} v=\{v^{1},v^{2},\cdots ,v^{G^{B}}\}, \end{array}$$

where $v^{g}=\left[v_{i}^{g}\right]\left(g=1,2,\cdots ,G^{B},i=1,2,\cdots ,t_{g}\right)$, $G^{B}$ is the number of groups and $t_{g}$ is the size of $v^{g}$.

Finally, the proposed JSEXIT algorithm for the D-LDPC codes system over AWGN channel is described as follows.

**Step 1: The MI update from VNs to CNs**

For $1\leq j\leq n_{s}$ and $1\leq i\leq m_{s}+m_{c}$

(17) $$\begin{array}{c} I_{ij,(k)}^{Ev}=\Omega \left(b_{ij}\right)J_{BSC}\left(\sum_{s\neq i}b_{is}{\left[J^{-1}\left(I_{sj,(k)}^{Av}\right)\right]}^{2}+\left(b_{ij}-1\right){\left[J^{-1}\left(I_{ij,(k)}^{Av}\right)\right]}^{2},\eta \right). \end{array}$$

The function $J_{BSC}$ is defined as [2]

$$\begin{array}{c} J_{BSC}\left(\mu ,\eta \right)=\left(1-\eta \right)I\left(V;\chi^{\left(1-\eta \right)}\right)+\eta I\left(V;\chi^{\eta }\right), \end{array}$$

where I(V;χ) is an MI calculation between the VN of the source and the distribution χ.

Further, $\chi^{\left(1-\eta \right)}∼N\left(\mu +Z_{v}^{sc},2\mu \right)$ and $\chi^{\eta }∼N\left(\mu -Z_{v}^{sc},2\mu \right)$ with $Z_{v}^{sc}=\ln\left(\left(1-\eta \right)/\eta \right)$.

For $\left(n_{s}+1\right)\leq j\leq \left(n_{s}+n_{c}\right)$ and $1\leq i\leq (m_{s}+m_{c})$

(18) $$\begin{array}{c} I_{ij,(k)}^{Ev}=\Omega \left(b_{ij}\right)J\left(\sqrt{\sum_{s\neq i}b_{sj}{\left[J^{-1}\left(I_{sj,(k)}^{Av}\right)\right]}^{2}+\left(b_{ij}-1\right){\left[J^{-1}\left(I_{ij,(k)}^{Av}\right)\right]}^{2}+\sigma_{ch,j}^{2}}\right). \end{array}$$

For $1\leq j\leq (n_{s}+n_{c})$ and $1\leq i\leq (m_{s}+m_{c})$

(19) $$\begin{array}{c} I_{ij,(k)}^{Ac}=I_{ij,(k)}^{Ev}. \end{array}$$

**Step 2: The MI update from CNs to VNs**

For $1\leq g\leq G^{B}$, $T_{g-1}\leq j\leq T_{g-1}+t_{g}$ and $1\leq i\leq (m_{s}+m_{c})$

(20) $$\begin{array}{c} I_{ij,(k)}^{Ec}=\Omega \left(b_{ij}\right)\left(1-J\left(\sqrt{\alpha_{\left(k\right)}+\alpha_{\left(k-1\right)}}\right)\right) \end{array}$$

where

(21) $$\begin{array}{c} \alpha_{\left(k\right)}=\left(\sum_{s\in \theta \left(i\right)\backslash j,\;s⩽T_{g-1}}b_{is}{\left[J^{-1}\left(1-I_{is,(k)}^{Ac}\right)\right]}^{2}\right), \end{array}$$

(22) $$\begin{array}{c} \alpha_{\left(k-1\right)}=\left(\sum_{s\in \theta \left(i\right)\backslash j,\;s>T_{g-1}}b_{is}{\left[J^{-1}\left(1-I_{is,(k-1)}^{Ac}\right)\right]}^{2}\right)+\left(b_{ij}-1\right){\left[J^{-1}\left(1-I_{ij,(k-1)}^{Ac}\right)\right]}^{2}, \end{array}$$

and set $I_{ij,(k)}^{Av}=I_{ij,(k)}^{Ec}$. Further, θ(i)\j denotes all VNs connected to the *i*-th CN excluding the *j*-th VN, $T_{g-1}$ is the size of the sum of the former sets $\left\{v^{1},v^{2},\cdots ,v^{g-1}\right\}$. It is noted that in the calculation of $I_{ij,(k)}^{Ec}$, partial $I_{is,(k)}^{Ac}$ has been updated to replace the $I_{is,(k-1)}^{Ac}$, which is reflected in the different calculations of $\alpha_{\left(k-1\right)}$ and $\alpha_{\left(k\right)}$.

**Step 3: The APP-LLR MI evaluation**

For $1\leq j\leq n_{s}$ and $1\leq i\leq m_{s}$

(23) $$\begin{array}{c} I_{j,(k)}^{APP}=J_{BSC}\left(\mu \left(j\right),\eta \right) \end{array}$$

where $\mu \left(j\right)=\sum_{i}b_{ij}{\left[J^{-1}\left(I_{ij}^{Av}\right)\right]}^{2}$.

The procedure of Steps 1 to 3 is performed iteratively until $I_{j}^{APP}=1$ or the maximum iteration is reached.

**Remarks**: If the maximum iteration is set to a large value, like the conventional EXIT algorithm, then the JSEXIT algorithm cannot reflect its advantage of shuffled scheduling, as with the larger iteration number in shuffled scheduling decoding, which has similar performance to that of the standard BP algorithm. We set the maximum iteration to 20 here for this reason. Therefore, the decoding threshold has a gap compared with that of the conventional EXIT algorithm, but it can provide comparable results.

### 4.2. Decoding Threshold Calculation

The MI $I_{j}^{APP}$ can be viewed as a function of independent variables $B_{J}$, η, $\sigma_{ch}$ and $G^{B}$, i.e.,

(24) $$\begin{array}{c} I_{j}^{APP}=\Upsilon \left(B_{J},\eta ,\sigma_{ch},G^{B}\right), \end{array}$$

where $\sigma_{ch}$ can be calculated from $E_{s}/N_{0}$. The channel decoding threshold ${\left(E_{s}/N_{0}\right)}_{th}$ indicates the performance of the water-fall region, which is the minimum value to make all $I_{j}^{APP}$ 1 for a given η. The $\eta_{th}$ indicates the performance of the error floor level, which is the maximum value to make all $I_{j}^{APP}$ 1 when $E_{s}/N_{0}\to \infty$. The ${\left(E_{s}/N_{0}\right)}_{th}$ and $\eta_{th}$ will also be calculated when a different $G^{B}$ is set. Without loss of generality, two examples using regular LDPC codes as source and channel code are presented as follows, where $B_{J1}$ is with $B_{l2}=0$ and another $B_{J2}$ is with $B_{l2}\neq 0$. The regular source and channel protographs with degree-3 VNs are given by

(25) $$\begin{array}{c} B_{s}^{reg}=B_{c}^{reg}=\left[\begin{array}{cccccccc} 1 & 0 & 1 & 1 & 1 & 0 & 1 & 1\\ 0 & 1 & 1 & 1 & 0 & 1 & 1 & 1\\ 1 & 1 & 0 & 1 & 1 & 1 & 1 & 0\\ 1 & 1 & 1 & 0 & 1 & 1 & 0 & 1 \end{array}\right]. \end{array}$$

The $B_{l1}$ is given by

(26) $$\begin{array}{c} B_{l1}=\left[\begin{array}{cccccccc} 1 & 0 & 0 & 0 & 0 & 0 & 0 & 0\\ 0 & 1 & 0 & 0 & 0 & 0 & 0 & 0\\ 0 & 0 & 1 & 0 & 0 & 0 & 0 & 0\\ 0 & 0 & 0 & 1 & 0 & 0 & 0 & 0 \end{array}\right]. \end{array}$$

The nonzero $B_{l2}^{nz}$ is represented by

(27) $$\begin{array}{c} B_{l2}^{nz}=\left[\begin{array}{cccccccc} 1 & 0 & 0 & 0 & 0 & 0 & 0 & 0\\ 0 & 1 & 0 & 0 & 0 & 0 & 0 & 0\\ 0 & 0 & 0 & 0 & 0 & 0 & 0 & 0\\ 0 & 0 & 0 & 0 & 0 & 0 & 0 & 0 \end{array}\right]. \end{array}$$

Thus, the $B_{J1}$ and $B_{J2}$ are respectively represented by

(28) $$B_{J1}=\left[\begin{array}{cc} B_{s}^{reg} & B_{l1}\\ 0 & B_{c}^{reg} \end{array}\right],\;B_{J2}=\left[\begin{array}{cc} B_{s}^{reg} & B_{l1}\\ B_{l2}^{nz} & B_{c}^{reg} \end{array}\right].$$

The source decoding thresholds $\eta_{th}$ and channel decoding thresholds ${\left(E_{s}/N_{0}\right)}_{th}$ for different grouping strategies are calculated and shown in Table 2 and Table 3. It can be seen that 1) the JSEXIT algorithm can calculate the decoding threshold regardless of the $B_{l2}=0$ or $B_{l2}\neq 0$; 2) with the increase of $G^{B}$, the source coding threshold becomes large and the channel threshold becomes small. For example, with $B_{J2}$ at source statistic η=0.11, the case of $G^{B}=16$ outperforms the cases of $G^{B}=8$, $G^{B}=4$, $G^{B}=2$ and $G^{B}=1$ by 0.12 dB, 0.35 dB, 1.17 dB and 1.27 dB, respectively.

## 5. Simulation and Comparison

In this section, we will illustrate the advantages of the JGSSD algorithm through Monte Carlo simulations and analyses of iteration number and decoding latency. For all simulations, the length of the source sequence is 3200, so the lifting factor of the PEG algorithm for $B_{J1}$ and $B_{J2}$ is 800. The maximum number of decoding iterations *K* is set as 30.

### 5.1. BER Performance

**Figure 2:**
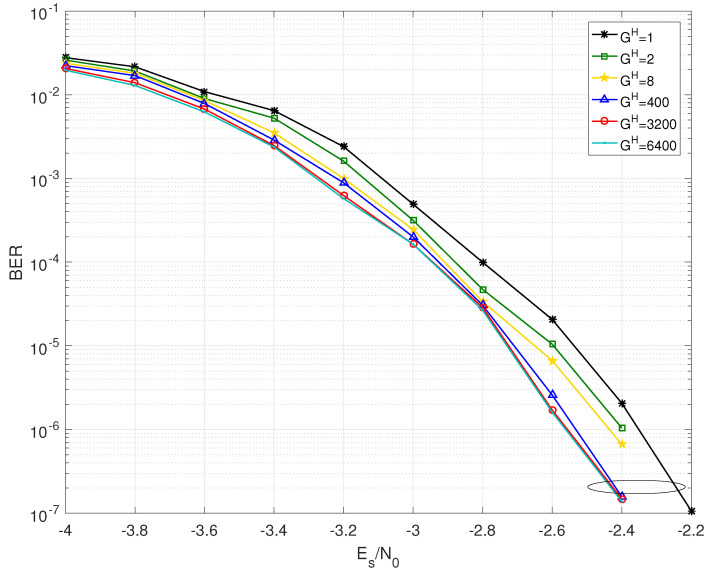
BER performance of $B_{J1}$ with $G^{H}=1,2,8,400,3200,6400$ at source statistic η=0.04.

**Figure 3:**
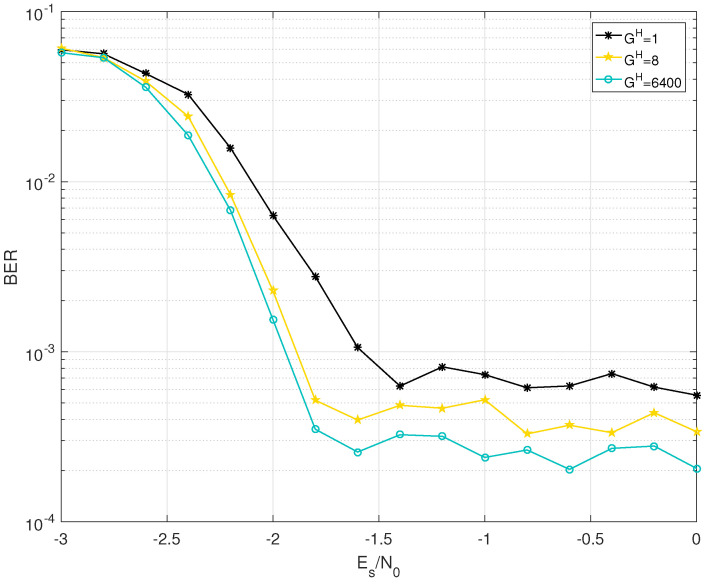
BER performance of $B_{J1}$ with $G^{H}=1,8,6400$ at source statistic η=0.07.

**Figure 4:**
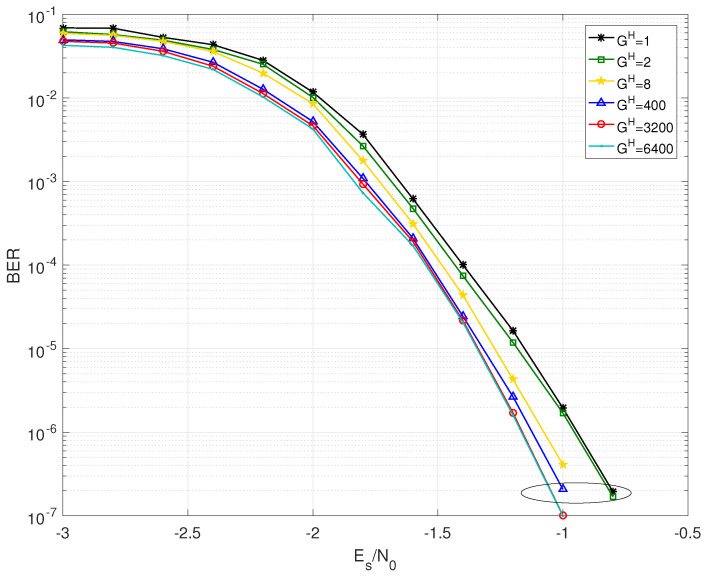
BER performance of $B_{J2}$ with $G^{H}=1,2,8,400,3200,6400$ at source statistic η=0.07.

**Figure 5:**
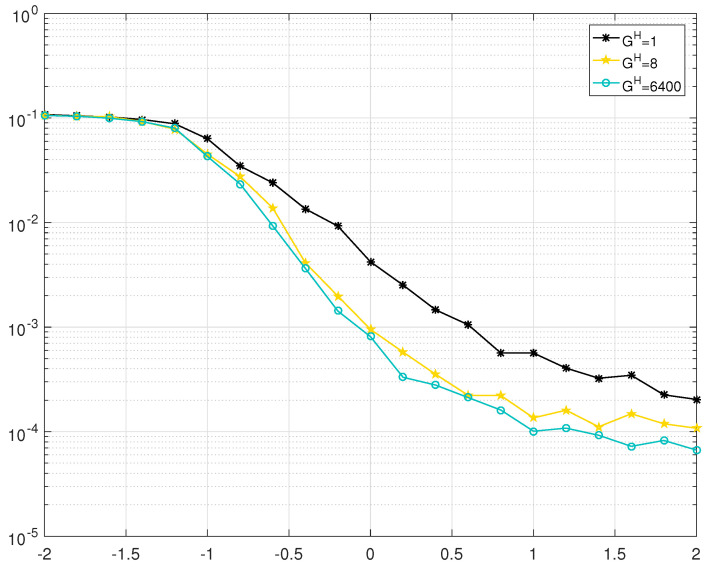
BER performance of $B_{J2}$ with $G^{H}=1,8,6400$ at source statistic η=0.11.

The BER performance of $B_{J1}$ and $B_{J2}$ with different grouping strategies are shown in Figure 2, Figure 3, Figure 4 and Figure 5. Firstly, it should be noted that the JGSSD algorithm is suitable for both the cases of $B_{l2}=0$ and $B_{l2}\neq 0$. Secondly, the BER performance in the water-fall region becomes better as the $G^{H}$ increases; this is in line with the EXIT analysis. It should be noted that the case of $G^{H}=3200$ is equivalent to the SSSD algorithm in [7,8]. For the case of $B_{J1}$ at source statistic at η=0.04, the case of $G^{H}=400$ has a coding gain of 0.2 dB compared with the case of $G^{H}=1$ and has no significant difference compared with $G^{H}=3200$ and $G^{H}=6400$ at the BER of $2\times 10^{-7}$. Other comparisons can be seen in Table 4. Thirdly, the error floor level also becomes lower as the $G^{H}$ increases, and this is in line with the EXIT analysis. For the case of $B_{J2}$ at source statistic at η=0.11, the case of $G^{H}=8$ is better than that of $G^{H}=1$, but worse than that of $G^{H}=6400$. It should be explained that the case $G^{H}=400$ and $G^{H}=3200$ have almost the same error floor level as that of $G^{H}=6400$.

### 5.2. Decoding Complexity and Latency

**Figure 6:**
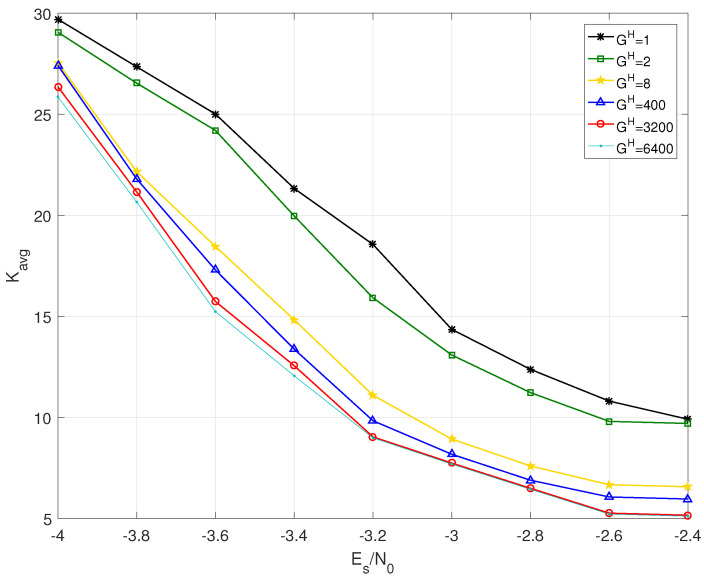
Average iteration number $K_{avg}$ of $B_{J1}$ with $G^{H}=1,2,8,400,3200,6400$ at source statistic η=0.04.

Without loss of generality, the case of $B_{J1}$ at source statistic η=0.04 is taken to compare the decoding complexity and latency. The decoding complexity can be evaluated by the decoding iteration number. Thus, the average iteration number $K_{avg}$ for different grouping strategies is shown in Figure 6. With increasing $E_{s}/N_{0}$, the $K_{avg}$ decreases, but the trend is getting smaller. The larger $G^{H}$ has a smaller $K_{avg}$ than that of the smaller $G^{H}$. For example, the cases of $G^{H}=400$ and $G^{H}=8$ respectively decrease by 40% and 34% compared with the case of $G^{H}=1$ at $E_{s}/N_{0}=-2.4$ dB, as shown in Table 5.

The decoding latency indicates the average time taken for information bits to be decoded. The decoding procedure consists of the C2V and V2C, and the total number of C2V and V2C depends on the average degree of CNs and VNs. Considering the same $B_{J1}$ used here, the decoding time of one group of C2V and V2C is the same, denoted as $t_{uni}$. Because the shuffled scheduling decoding algorithm is serial but the BP decoding algorithm is parallel, different grouping strategies imply the combination of serial and parallel methods. Take the case $G^{H}=400$ as an example: 400 groups perform the decoding procedure one by one, and every group of these 400 groups performs parallel decoding. Thus, the process will consume 400 $t_{uni}$ in an iteration. If the average iteration is $K_{avg}$, the average time of decoding information bits can be calculated by

(29) $$\begin{array}{c} T_{avg}=t_{uni}\times G^{H}\times K_{avg}. \end{array}$$

As shown in Table 5, the decoding latency increases with the increase of $G_{H}$.

A reasonable decoding solution, i.e., the grouping strategy, should take BER performance, decoding complexity and decoding latency into consideration.

## 6. Conclusions

In this paper, a JGSSD algorithm for the D-LDPC codes system is proposed. The proposed algorithm considers the D-LDPC coding structure as a whole. In order to analyze the performance of different grouping strategies, a JSEXIT algorithm is proposed for the general D-LDPC coding structure, i.e., both the cases of $B_{l2}=0$ and $B_{l2}\neq 0$. It can be seen that both the source decoding threshold and the channel decoding threshold improve as $G^{B}$ increases. The BER simulation is in line with the EXIT analysis, i.e., the BER performance has a better coding gain or lower error floor level when the $G^{H}$ has a higher value. In addition, the decoding complexity and decoding latency are also compared, and it is shown that the larger $G^{H}$ gives a lower decoding complexity but a higher decoding latency. Thus, a suitable shuffled scheduling decoding algorithm should give overall consideration to factors including performance, complexity and latency, and the JGSSD algorithm provides an intelligent choice. In future, the performance of the JGSSD algorithm can be studied for specific applications, such as multiple fading channels, and the optimization of the D-LDPC coding structure with the aid of the JSEXIT algorithm for different grouping strategies can be studied.

## Figures and Tables

**Table 1:** The main differences between the present work (JGSSD) and previous work (IBP and SSSD).

	IBP (e.g., [2])	SSSD [7,8]	JGSSD
Decoding order	Parallel	Serial	Parallel and Sequential
EXIT algorithm	∖	only for algorithm SSSD with the case $B_{l2}=0$	both for the SSSD and JGSSD with the case $B_{l2}=0$ and $B_{l2}\neq 0$
Complexity	High	Low	Adaptive
Latency	Low	High	Adaptive
Adaptive judgement	No	No	Yes

**Table 2:** Source decoding thresholds $\eta_{th}$ and channel decoding thresholds for ${\left(E_{s}/N_{0}\right)}_{th}$ at η=0.04 and 0.07 for $B_{J1}$ with different grouping strategies.

$B_{J1}$	$\eta_{\mathrm{th}}$	${\left(E_{s}/N_{0}\right)}_{\mathrm{th}}$	
		0.04	0.07
$G^{B}=1$	0.072	−3.13 dB	−1.39 dB
$G^{B}=2$	0.073	−3.27 dB	−1.41 dB
$G^{B}=4$	0.077	−3.46 dB	−2.00 dB
$G^{B}=8$	0.078	−3.54 dB	−2.19 dB
$G^{B}=16$	0.079	−3.58 dB	−2.25 dB

**Table 3:** Source decoding thresholds $\eta_{th}$ and channel decoding thresholds for ${\left(E_{s}/N_{0}\right)}_{th}$ at η=0.07 and 0.11 for $B_{J2}$ with different grouping strategies.

$B_{J2}$	$\eta_{\mathrm{th}}$	${\left(E_{s}/N_{0}\right)}_{\mathrm{th}}$	
		0.07	0.11
$G^{B}=1$	0.118	−1.67 dB	0.65 dB
$G^{B}=2$	0.119	−1.77 dB	0.55 dB
$G^{B}=4$	0.125	−1.98 dB	−0.27 dB
$G^{B}=8$	0.128	−2.07 dB	−0.50 dB
$G^{B}=16$	0.129	−2.10 dB	−0.62 dB

**Table 4:** $E_{b}/N_{0}$ Gain at BER = $1\times 10^{-6}$ and error floor levels for different grouping strategies.

Grouping Strategy	$E_{b}/N_{0}$ Gain at BER = $1\times 10^{-6}$		Error Floor Level	
	$\eta =0.04,B_{J1}$	$\eta =0.07,B_{J2}$	$\eta =0.07,B_{J1}$	$\eta =0.11,B_{J2}$
$G^{H}=1$	0	0	$7\times 10^{-4}$	$2\times 10^{-4}$
$G^{H}=2$	0.05 dB	0.01 dB	-	-
$G^{H}=8$	0.08 dB	0.12 dB	$5\times 10^{-4}$	$1\times 10^{-4}$
$G^{H}=400$	0.18 dB	0.18 dB	-	-
$G^{H}=3200$	0.19 dB	0.22 dB	-	-
$G^{H}=6400$	0.19 dB	0.22 dB	$3\times 10^{-4}$	$9\times 10^{-5}$

**Table 5:** The decoding complexity (average iteration number $K_{avg}$) and the decoding latency (average decoding time $T_{avg}$) for different grouping strategies of $B_{J1}$ at η=0.04 and $E_{s}/N_{0}=-2.4$ dB.

Grouping Strategy	$K_{\mathrm{avg}}$	$T_{\mathrm{avg}}$
$G^{H}=1$	9.9	$9.9t_{uni}$
$G^{H}=2$	9.7	$19.4t_{uni}$
$G^{H}=8$	6.5	$52t_{uni}$
$G^{H}=400$	5.9	$2360t_{uni}$
$G^{H}=3200$	5.1	$16,320t_{uni}$
$G^{H}=6400$	5.1	$32,640t_{uni}$

## Abbreviations

The following abbreviations are used in this manuscript:

AWGN	additive white Gaussian noise
APP	a posteriori probability
BER	bit error rate
BP	belief propagation
CNs	check nodes
C2V	check-to-variable
D-LDPC	double low-density parity-check
EXIT	extrinsic information transfer
IBP	iterative belief propagation
IoT	Internet of things
JBP	joint belief propagation
JEXIT	joint extrinsic information transfer
JPEXIT	joint protograph EXIT
JGSSD	joint group shuffled scheduling decoding
JSCC	joint source-channel coding
JSEXIT	joint shuffled EXIT
LLR	log-likelihood-ratio
MI	mutual information
PEG	progressive edge growth
SPEXIT	source protograph EXIT
SSSD	separated shuffled scheduling decoding
SWD	sliding window decoding
V2C	variable-to-check
VNs	variable nodes
